# The effects of being told you are in the intervention group on training results: a pilot study

**DOI:** 10.1038/s41598-023-29141-7

**Published:** 2023-02-03

**Authors:** Kolbjørn Lindberg, Thomas Bjørnsen, Fredrik T. Vårvik, Gøran Paulsen, Malene Joensen, Morten Kristoffersen, Ole Sveen, Hilde Gundersen, Gunnar Slettaløkken, Robert Brankovic, Paul Solberg

**Affiliations:** 1 Department of Sport Science and Physical Education, Faculty of Health and Sport Sciences, University of Agder, Kristiansand, Norway; 2 Norwegian Olympic Federation, Oslo, Norway; 3 Department of Physical Performance, Norwegian School of Sport Sciences, Oslo, Norway; 4 Department of Health and Functioning, Western Norway University of Applied Sciences of Norway, Bergen, Norway; 5 Department of Sport, Food and Natural Sciences, Western Norway University of Applied Sciences, Bergen, Norway; 6 Østfold University College, Halden, Norway

**Keywords:** Physiology, Psychology

## Abstract

Little is known about the placebo effects when comparing training interventions. Consequently, we investigated whether subjects being told they are in the intervention group get better training results compared to subjects being told they are in a control group. Forty athletes (male: n = 31, female: n = 9) completed a 10-week training intervention (age: 22 ± 4 years, height: 183 ± 10 cm, and body mass: 84 ± 15 kg). After randomization, the participants were either told that the training program they got was individualized based on their force–velocity profile (Placebo), or that they were in the control group (Control). However, both groups were doing the same workouts. Measurements included countermovement jump (CMJ), 20-m sprint, one-repetition maximum (1RM) back-squat, a leg-press test, ultrasonography of muscle-thickness (m. rectus femoris), and a questionnaire (Stanford Expectations of Treatment Scale) (Younger et al. in Clin Trials 9(6):767–776, 2012). Placebo increased 1RM squat more than Control (5.7 ± 6.4% vs 0.9 ± 6.9%, [0.26 vs 0.02 Effect Size], Bayes Factor: 5.1 [BF_10_], p = 0.025). Placebo had slightly higher adherence compared to control (82 ± 18% vs 72 ± 13%, BF_10_: 2.0, p = 0.08). Importantly, the difference in the 1RM squat was significant after controlling for adherence (p = 0.013). No significant differences were observed in the other measurements. The results suggest that the placebo effect may be meaningful in sports and exercise training interventions. It is possible that ineffective training interventions will go unquestioned in the absence of placebo-controlled trials.

## Introduction

The placebo effect describes a favorable outcome that occurs because of one's belief or expectation that one has received a positive intervention^2^. Given the prevalence of placebo effects, researchers across a wide range of disciplines have attempted to control them for nearly 80 years, with the first placebo-controlled clinical trial published in 1944^3,4^. Similarly, over the last two decades, research in sport and exercise science has shown that placebo and nocebo effects can have a major impact on athletic performance^5^.

Notably, studies investigating the placebo effect in sports science are conducted with placebo dietary supplements such as caffeine, creatine monohydrate, carbohydrate, and even anabolic steroids^6^. When the treatment is administered in the form of tablets, injections, capsules, or other comparable forms, studying the effects of a placebo is relatively simple^6^. However, from medicine, we know that the effectiveness of placebos can vary with the administration form^7^. For example, sham surgeries have been shown to induce very strong placebo effects^7^, and placebo injections exhibit stronger effects than placebo pills^8^. Following recent advances in placebo research, several forms of interventions have indeed been challenged due to the possibility of strong placebo effects being present^3^. In sports science, we are frequently comparing the efficacy of different resistance training interventions (e.g. comparing exercise selection, loading schemes, frequency, or volume), where it is very difficult to control for the placebo effect^5^. Consequently, it is likely that previous research findings in sport and exercise science are confounded by placebos^5^. As noted by several authors, most training interventions are unable to administer placebos due to obvious reasons such as blinding (i.e., we cannot tell subjects that they are lifting weights 3 days a week, if they are indeed lifting 6 days a week)^2,5,6^. More importantly, most previous training studies do not mention nor control for the participant's or researchers' expectations towards the treatment^2,5,6^. It is well known that the researcher's and/or participants' expectations of the intervention can have a major impact on the study's outcome^9–11^. As a result, it is probable that the advocates/inventors of new training approaches will find the efficacy of such concepts to be slightly more effective because of placebo effects^9–11^.

A recent popular training concept within sports science is training according to participants' individual "force–velocity" (FV) profiles. Briefly, the individualized training is theorized to work by changing athletes' force–velocity profiles towards a theoretical optimal profile^12^. In practice, athletes with a "force-oriented profile" (i.e., velocity deficit) are commonly prescribed training with a focus on high-velocity exercises, whereas athletes with "velocity-oriented profiles" (i.e., force deficit) get prescribed high-force exercises. Athletes with a "well-balanced" profile, then get training prescriptions with a balanced combination of both high-force and high-velocity training^13–20^. When training according to the FV profiles, the subjects get "individualized" training based on a performance test. The performance test that determines which form of training is "optimal" is a "black box" for the participants, which makes it a perfect setup to investigate the placebo effect (i.e., participants can easily be randomized and told they get optimal or control training without knowing which is actually "optimal")^13–20^. Therefore, in practice, two participants can be doing the exact same workouts, but it is "optimal" for one subject and "non-optimal" for another. The concept is found to be highly effective in some studies, while other studies have yielded different findings^13–20^.

Currently, we know very little about the potential placebo effect when investigating different training configurations (e.g., exercise selection, loading, volume, frequency). Hence, the present study aimed to investigate whether a placebo effect is present when participants are told they get "optimal training" compared to being told they get generic "control training".

## Methods

### Experimental design and participants

Seventy-one athletes were recruited for the study. The participants first completed baseline assessments; a 10-week training intervention followed by post-intervention tests. Due to the Covid-19 pandemic, multiple participants either got sick or quarantined during the study period and were unable to either complete the intervention/or testing sessions. Details regarding the number of dropouts in each group are presented in the CONSORT diagram. Additionally, group comparisons for the dropouts are presented in the results section. The adherence to the training program is also reported in the results section. The number of participants referred to throughout the manuscript is the participants completing the training intervention and pre- and post-testing (n = 40). The athletes were national and club level team sport players in handball (males, n = 31), and soccer (females n = 9), with an average age of 22 ± 4 years, height of 183 ± 10 cm, and body mass of 84 ± 15 kg. The data were collected from multiple regional Olympic training and testing centers. Prior to participation, written informed consent was obtained. The study was approved by the ethical board of the University of Agder's faculty of health and sports science, as well as the Norwegian Centre for Research Data, and was carried out in accordance with the Declaration of Helsinki (except pre-registration). The subjects had to be healthy and not taking any medication that could interfere with the study. All subjects had to be familiar with strength training with a minimum of 6 month of practice. Due to the relatively small sample size, the present study should be considered a pilot study.

**Figure 1:**
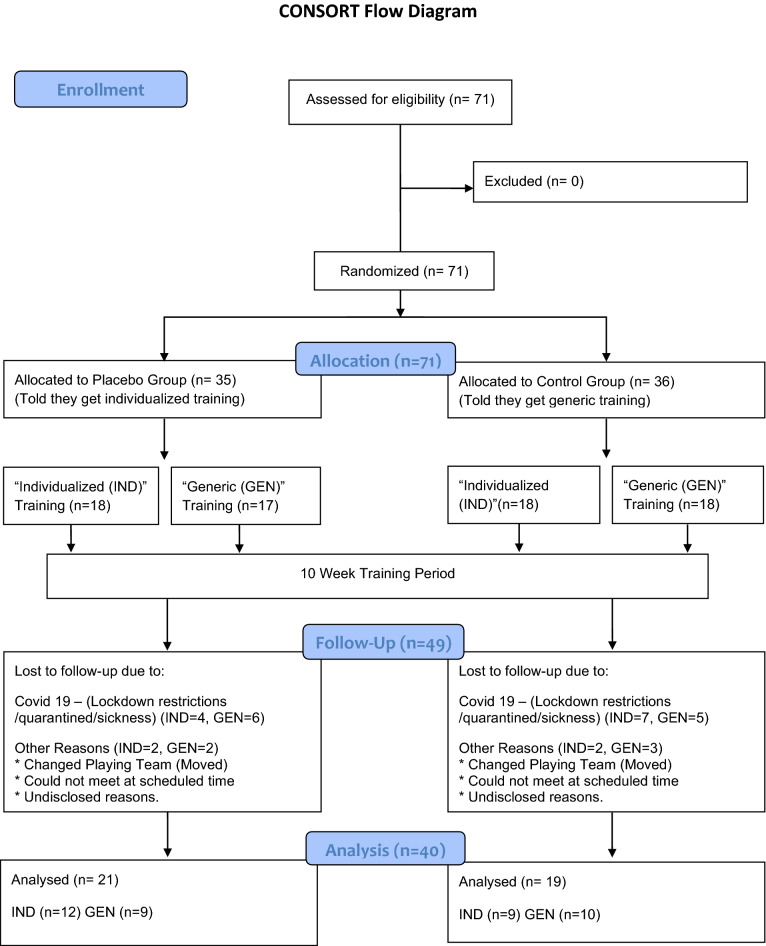
Flow chart of the study design.

The participants were first randomly assigned to one of two groups, Placebo, or Control. In each of these groups, they were again randomized to either a generic power training program or an individualized training program based on their individual force–velocity profile. See Fig. 1 for study design illustration.

### Administration of placebo

To administer the placebo treatment, the participants were either told that the training program they got was individualized based on their force–velocity profile, or that they were in the control group. This means, that both groups consisted of subjects doing the same workouts, but half of them believed they did optimal individualized training (Placebo), and the other half believed they were the control group with non-optimal generic training (Control) (Fig. 1). Importantly, as the baseline FV profiles were calculated by a researcher that did not participate in testing or training of the athletes, the FV profile was unknown to the participants and researchers involved in measurements and training. Therefore, administration of the Placebo was possible by telling some of the subjects they have another profile than what is measured. For example, subjects in the Placebo group who got the "force focused training", were all told their FV profiles were velocity-oriented (force "deficit"), and that heavy load training was the optimal training for them. In contrast, participants in the Control group did not receive any information regarding their FV profiles. They were told that they were in the control group, and that the training program they received was developed to improve performance without individualizing based on FV-profiles. All the participants got these instructions verbally as well as in written format. The training programs and exact instructions given to the participants are added as supplementary material (Supplementary material 1). An overview of the training program is presented in Table 1. The training sessions were not supervised by the research team. Exercises were performed in the order they are written in the supplementary Tables.

**Table 1:** Training content for the 3 different training programs.

	Exercises	Rep scheme	Load	Weekly sets	Focus	% of sets
Force program	Deadlift, Hip-thrust, Front squat, Squat, Stiff-leg dead lift, Bulgarian split squat, Trapbar Deadlift, Calf-raises	3–10	1–6 RIR	14	Strength	82
	Trapbar Deadlift	5	50–70% 1RM	4	Power	18
Balanced program	Deadlift, Front squat, Bulgarian split squat, Hip-thrust, Deadlift	3–10	1–6 RIR	13	Strength	46
	Box jumps, Stair jumps, Single leg stair jumps, Squat jump w/rubber band, Stair jumps, Trapbar jumps	5–10	Negative-50% 1RM	15	Power	54
Velocity program	Half Squat, Hip-thrust	3–8	1–2 RIR	6	Strength	21
	Squat jumps, Trapbar jumps, Step up, Squat jump w/rubber band, countermovement jumps, box jumps, Clean Pull, Stair jumps, Single leg stair jumps	5–10	Negative-50% 1RM	22	Power	79

*RIR* reps in reserve, *1RM* one repetition maximum, *reps* repetitions, *Set* training sets.

### Testing procedures

All subjects were told to prepare for the test days in the same way as for a regular competition in terms of nutrition, hydration, and sleep, and to avoid excessive exercise 48 h before the test. Before testing, participants completed a standardized 10-min warm-up that included jogging, local muscle warm-up (hamstring and hip mobility), running drills (such as high knees, skipping, butt-kicks, and explosive lunges), and body mass jumps. The testing protocol included a series of countermovement jumps (CMJ's) with incremental loads, 20-m sprints, 1RM back-squat, and leg-press tests. All the subjects were provided with verbal encouragement and instructions to help them do their very best on the performance tests. It is however important to note that during the testing, neither the individuals nor the people in charge of the test were aware of the group allocation. Ultrasound measures were taken before the physical testing, or on a separate test day for some of the participants.

The ultrasound measurements were conducted using a brightness mode (B-mode) ultrasonography device (Telemed ArtUS EXT-1H, IT, 70 Hz, Vilnius, Lithuania, EU) using a 60-mm probe (LV8-5N60-A2) measuring resting muscle thickness of m. rectus femoris. All participants lay supine on an examination bench with knees fully extended. The measurement was taken at ~ 40% from the lateral epicondyle of the knee to the great trochanter major^20^. Ultrasound settings (Gain, frequency, depth) were optimized for each subject and kept constant at each test session, to best highlight collagenous tissue that constitutes muscle aponeuroses and surrounds muscle fascicles. A transparent sheet was used to record the scanning location relative to natural landmarks such as scars, moles, birthmarks etc. All ultrasound pictures were analyzed using ImageJ (version 1.46r, National Institutes of Health, USA), in a blinded manner (i.e. not the same examiner who took the pictures, and also blinded for the group allocation). The ultrasound measures were taken from 1 picture per subject. Based on pilot testing, we found this procedure to have < 3% test–retest variation.

A modified version of the Stanford Expectations of Treatment Scale (SETS) was utilized to examine expectancy effects. The SETS scale is a previously validated tool for assessing positive and negative treatment expectations in clinical trials^1^. The questionnaire was translated to Norwegian where some of the questions were excluded to make it easier to administer to the participants. All the questionnaires collected from the participants were double-checked to see if any were missing or incomplete. Those that were left blank, patterned, or all marked the same choice, were excluded from the analyses (< 3% of answers). Each participant was instructed to take note of each completed training session to control adherence to the training program. At the post-test—the participant reported their number of completed sessions together with the SETS questionnaire^1^. In accordance with previous research, the adherence was then reported as percentages (i.e., % completed sessions of scheduled sessions).

The CMJ's were performed with an incremental loading protocol of 3 loads, starting at bodyweight, increasing to 40 kg, and the last load was individually adjusted with a goal of jumping approximately 10 cm (range 60–90 kg). The subjects performed 2–3 jumps × 2 for the bodyweight and 40 kg condition and 1–2 jumps × 2 for the heaviest load. The rest between jumps within sets were approximately 10–20 s and about 2–3 min between sets and loads. For all the jumps, the CMJ- depth was standardized to the athletes' self-selected starting position, controlled visually and by the displacement output from the force-plate software. The jump height was measured with a force plate sampling at 1000 Hz (AMTI; Advanced Mechanical Technology, Inc, Waltham Street, Watertown, USA or; Kistler 9286B force plate, Kistler Instruments AG) and calculated from the impulse. The average of the best two trials for each jump condition were used for further analysis. To calculate the actual and optimal FV profile, the proposed methods of Samozino et al.^12^ were used. Based on the jump height, body mass, and push-off distance of the subjects, average force and velocity were obtained. Followingly, a linear regression was fitted to the average force and velocity values, where Samozino's method was used to calculate the theoretical optimal FV profile^12^. The difference between the extended lower limb length with maximal plantar flexion and the crouch starting position of the jump was used to calculate the vertical push-off distance, as previously proposed^12^. The bodyweight of the subjects were measured from the steady stance at the force plate.

The participants performed 2–4 maximal sprints of 20-m, with 3–5 min of recovery in between each trial. The timing began when the front foot left the ground and was measured using wireless timing gates at 5-m intervals (Musclelab, Ergotest innovation AS, Langesund, Norway). For subsequent analysis, the best 20-m time was used.

The leg press was performed on a Keiser A300 horizontal leg-press dynamometer (Keiser Sport, Fresno, CA). The FV variables were determined using a 10-repetition FV test with incremental loads based on each participant's estimated 1RM load. The estimation of the 1RM load is based on the test leader’s subjective judgment and is considered to be a reliable method for accurately acquiring a FV-profile^21^. Each participant's seating posture was modified to achieve a vertical femur, which corresponded to an 80°–90° knee angle, and feet were placed with heals at the bottom end of the foot pedal. Throughout the 10-repetition FV test, participants were required to extend both legs with maximal effort. The test began with two practice attempts at the lightest load, corresponding to 15% of 1RM. As the load increased, the rest period between attempts increases. For the first five loads, the rest time was 10–20 s, while the last four rest periods were 20–40 s. Because the pedals were resting in their predetermined position before each repetition, the leg press was executed as a concentric-only action with no countermovement. The eccentric phase was not registered. The theoretical maximum power from the FV-profile was then used to calculate leg press power.

The 1RM back-squat was obtained using a standardized protocol, with incremental loading until 1RM was attained. Submaximal squats with 2–4 repetitions at 50% and 60% of 1RM were conducted as part of a brief warm-up, following one repetition at 80%, 90%, and 95% of 1RM (self-estimated at the first time-point). The participants were then given 2–3 trials at the 1RM load with a rest period after each attempt of 2–3 min. The minimum load increase was 2.5 kg and the heaviest load (in kg) successfully lifted with the standardized depth was recorded as the participant's 1RM. The test leaders visually validated that the squat depth was standardized to thighs parallel to the ground (the top surface of the legs at the hip joint is lower than the top of the knees). At all times during the study, the standardized squat depth was maintained^22^. The relative 1RM value (kg/bw) was used for further analysis, as this is closer related to common measures of athletic performance^23^.

### Statistical analyses

In combination with traditional null-hypothesis testing, a Bayesian approach was used because it is less dependent on sample size, compared with traditional p-values^24,25^. Given our multicenter study design, where the sample size for some of the measures was lower, a Bayesian approach was regarded as more robust^24,25^. The data were checked for normal distribution using the Shapiro–Wilk test before analysis. An independent sample *t* test was conducted to examine the differences between placebo and control groups for all the included measures, in addition to baseline differences between groups. Only the variables from the SETS scale were found to be non-normally distributed and is presented as median and quartiles and differences between groups were analyzed with a rank-biserial coefficient of correlation for the upper and lower quartiles. Additionally, an ANCOVA was conducted to control for potential confounding effects from the expectancy measures and the adherence of the subjects. A paired sample *t* test was used to examine changes within groups before and after the intervention. The standardized effect size (ES) was computed by dividing the pre-post changes by the pooled pre-SD (from all participants) and was categorized as (0.20–0.60 small; 0.60–1.20 moderate; 1.20–2.00 large; > 2 extremely large). Unless otherwise stated, means with the corresponding variance are shown with standard deviation (SD). The interpretation of the Bayes factor (BF_10_) follows the scale proposed by Jeffreys^26^ (1–3 anecdotal; 3–10 substantial; 10–30 strong; 30–100 very strong and > 100 decisive evidence for H_1_, whereas BF_10_ < 1 suggests support for H_0_). The significance level was set at 0.05 and the confidence level were set at 95% for all analyses. Statistical analyses were conducted using JASP version 0.14 (JASP 2020).

## Results

There were no significant baseline differences in any of the performance measures, between the placebo and control group (Table 2). There were no difference in age of the subjects between the two groups (Placebo: 22 ± 4y, Control: 22 ± 5y, p = 0.83). Further, there were no significant difference at baseline between the subjects that dropped out vs the subjects who completed the entire study.

**Table 2:** Results from the main groups, individualized (Placebo [PLA]) vs control group (Control [CON]).

Variable and group	Pre	Post	Change		Group difference			
	Mean ± SD	Mean ± SD	Δ ± SD	ES	Mean ± 95% CI	ES	BF_10_	p-value
1RM squat (kg/bw)								
Placebo (PLA)	1.61 ± 0.43	1.71 ± 0.45	0.10 ± 0.10***	0.26	*PLA vs CON:*			
Control (CON)	1.54 ± 0.29	1.54 ± 0.22	0.01 ± 0.10	0.02	0.09 ± 0.08	0.24	5.10	0.03*
10m sprint (s)								
Placebo (PLA)	1.61 ± 0.10	1.60 ± 0.11	− 0.01 ± 0.03	− 0.06	*PLA vs CON:*			
Control (CON)	1.63 ± 0.12	1.62 ± 0.12	− 0.01 ± 0.03	− 0.11	0.01 ± 0.02	0.05	0.24	0.67
20m sprint (s)								
Placebo (PLA)	2.92 ± 0.19	2.90 ± 0.19	− 0.03 ± 0.06	− 0.13	*PLA vs CON:*			
Control (CON)	2.95 ± 0.21	2.94 ± 0.19	− 0.01 ± 0.06	− 0.07	− 0.01 ± 0.04	− 0.06	0.52	0.50
CMJ Jump height (cm)								
Placebo (PLA)	38.2 ± 7.21	38.6 ± 7.2	0.4 ± 1.8	0.07	*PLA vs CON:*			
Control (CON)	34.1 ± 5.01	34.8 ± 5.5	0.7 ± 2.1	0.12	− 0.26 ± 1.54	− 0.04	0.27	0.80
Leg press power (w/bw)								
Placebo (PLA)	20.2 ± 3.3	20.0 ± 3.4	− 0.2 ± 1.3	− 0.07	*PLA vs CON:*			
Control (CON)	19.2 ± 2.8	18.9 ± 2.7	− 0.3 ± 1.1	− 0.09	0.06 ± 0.83	0.02	0.36	0.74
Muscle thickness (mm)								
Placebo (PLA)	23 ± 4.7	24.0 ± 5.4	1.0 ± 1.3#	0.24	*PLA vs CON:*			
Control (CON)	24 ± 3.8	24.2 ± 4.1	0.2 ± 2.0	0.05	0.78 ± 2.12	0.20	0.89	0.27

*1RM* 1-repetition maximum, *CMJ* countermovement jump, *kg* kilogram, *s* seconds, *cm* centimeters, *W* Watts, *mm* millimeters. ***p < 0.001, **p < 0.01, *p < 0.05.

**Figure 2:**
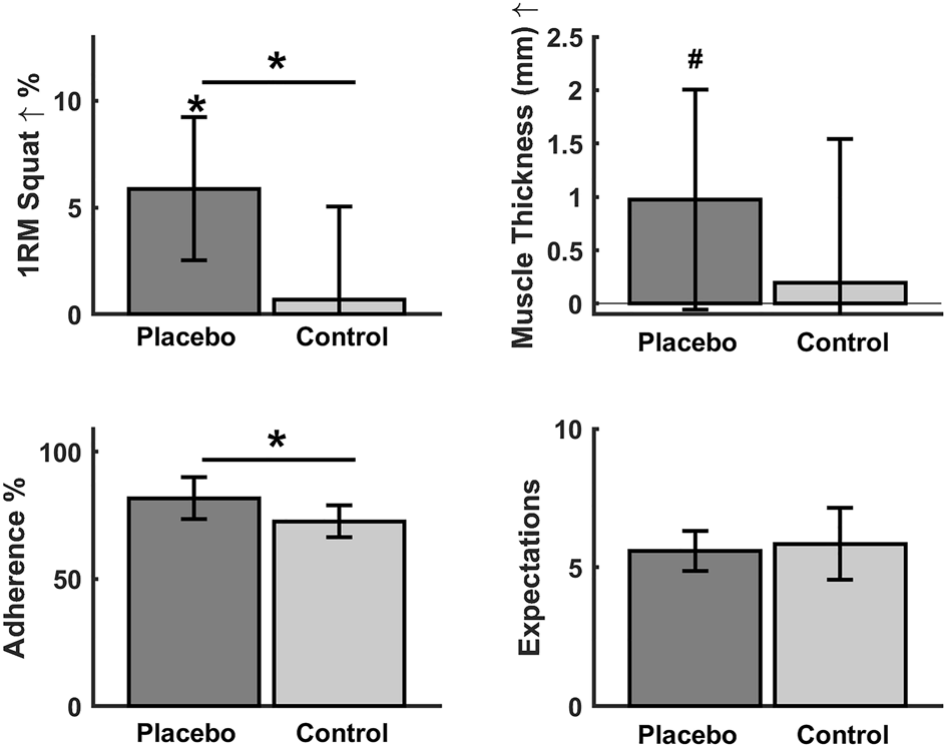
Illustrating percent change in 1RM (*One repetition maximum*) squat, change in muscle thickness (mm: millimeters), Adherence between groups measured as percentage of completed scheduled training sessions as well as median expectation (SETS: Stanford Expectations of Treatment Scale) of the placebo and control group. *p < 0.05 ^#^p < 0.10, where the horizontal line represent group changes. Error bars represent 95% confidence intervals (except for SETS which illustrate median with upper and lower quartiles).

Placebo increased 1RM squat more than Control (5.7 ± 6.4% vs 0.9 ± 6.9%, Bayes Factor: 5.1 [BF_10_], p = 0.025). Additionally, Placebo increased muscle-thickness compared to baseline (3.3 ± 6.1%, BF_10_: 3.0, p = 0.06), whereas there was no change from baseline in Control (− 1.9 ± 14.0%, BF_10_: 0.3, p = 0.89). Placebo had slightly higher adherence compared to the control group (placebo: 82 ± 18% control: 72 ± 13%, difference: BF_10_: 2.0, p = 0.08) (Fig. 2). The group difference in 1RM squat were significant after adjusting for adherence (F = 7.1, n^2^ = 0.19, p = 0.013), and the SETS expectation level (F = 5.4, n^2^ = 0.16, p = 0.027).

**Figure 3:**
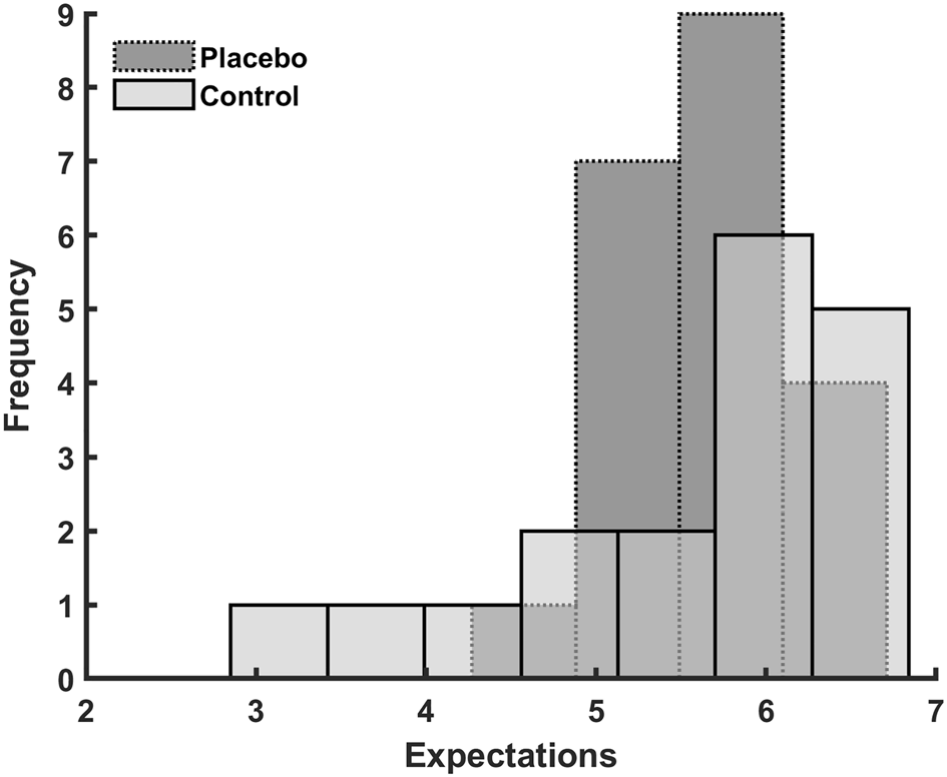
Frequency distribution of the expectations for the placebo vs control group.

No significant differences between groups were observed in CMJ, 20-m sprints or leg press power (Table 2). The expectations towards the intervention showed a moderate correlation with the adherence to the training (r = 0.39, BF_10_: 3.8, p = 0.013). Both groups reported similar median levels of expectations towards the interventions (Placebo: 5.6 ± 0.7 Control: 5.9 ± 1.1 [median and quartiles]). However, the expectations were not-normal distributed in Control, and the subjects in the lower quartile of Control had lower expectations towards the training intervention compared to Placebo (r = 0.72 [rank-biserial coefficient of correlation], BF_10_: 2.1, p = 0.033, Fig. 3). Additionally, there was a strong correlation between changes in muscle thickness and changes in 1RM squat (r = 0.58, BF_10_: 6.3, p = 0.025). There was no correlation between adherence and changes in 1RM squat (r = − 0.12, BF_10_: 0.2, p = 0.53). There were no changes in bodyweight in any of the groups (Placebo: − 0.2 ± 1.7 kg, p = 0.66, Control: 0.0 ± 0.9 kg, p = 0.96).

No significant differences were observed in any of the performance measures when comparing all participants doing the actual theoretical "optimal" individualized training vs participants performing generic power training. Notably, all participants in the «Individualized» training subgroups were deemed “velocity oriented” by the calculations from Samozino et al.^12^, and conducted high-load strength training.

## Discussion

The study's key finding was that, despite undergoing the same training, participants who were told they were in the intervention group (Placebo) improved their 1RM squat more than those in the control group (Control). Additionally, the subjects in the placebo group tended to increased muscle thickness, which was strongly correlated with changes in leg strength.

To the author's knowledge, this is the first study investigating the placebo effect as a consequence of altering participants' expectations of a training intervention. A recent review on the topic summarized the findings of placebo research in sports science and found a pooled effect size of 0.37, combining a variety of placebo treatments^6^. Further, the effects varied depending on the type of Placebo administered^6^. Nutritional and mechanical ergogenic aids both had small to moderate placebo effects (ES: 0.35–0.47)^6,27,28^. The effects of anabolic steroids as a placebo had the greatest impact on performance (ES: 1.44)^6,29,30^. The effects of a placebo evoked by an erythropoietin-like (EPO) drug on performance were likewise found to elicit large effects (ES: 0.81)^31^. The placebo effect of Transcutaneous Nerve Stimulation (TENS) was observed to have moderate to high effect sizes (ES: 0.70–1.02)^6,32,33^, whereas amino acids, caffeine, and placebo tennis rackets have small to moderate effect sizes (ES: 0.36–0.40)^6,34–36^. Fake sports supplements were shown to have small effects on performance (ES: 0.21)^37^. Coldwater immersion, sodium bicarbonate, ischemia preconditioning, carbohydrate, -alanine, kinesiology tape, and magnetic wristbands had no measurable effect^6,38–40^. The effect size in the present study (ES: 0.26) is comparable to the literature, where the fake sports supplements might be the studies with the most comparable effects and study designs. Specifically, the subjects are tested in physical performance measures, whereas some are told they get a performance-enhancing substance, and others get the same substance, but are told it does not increase performance. Notably, the main difference in our present study is the training duration, as most other placebo studies investigate acute measures^6^. Additionally, our emphasis was on the training configurations of the training program and not from a nutritional substance. Previous studies usually see larger strength gains in 10-week training periods (ES > 0.50), however as the present study were in season for the athletes, smaller effects would be expected^41,42^.

**Figure 4:**
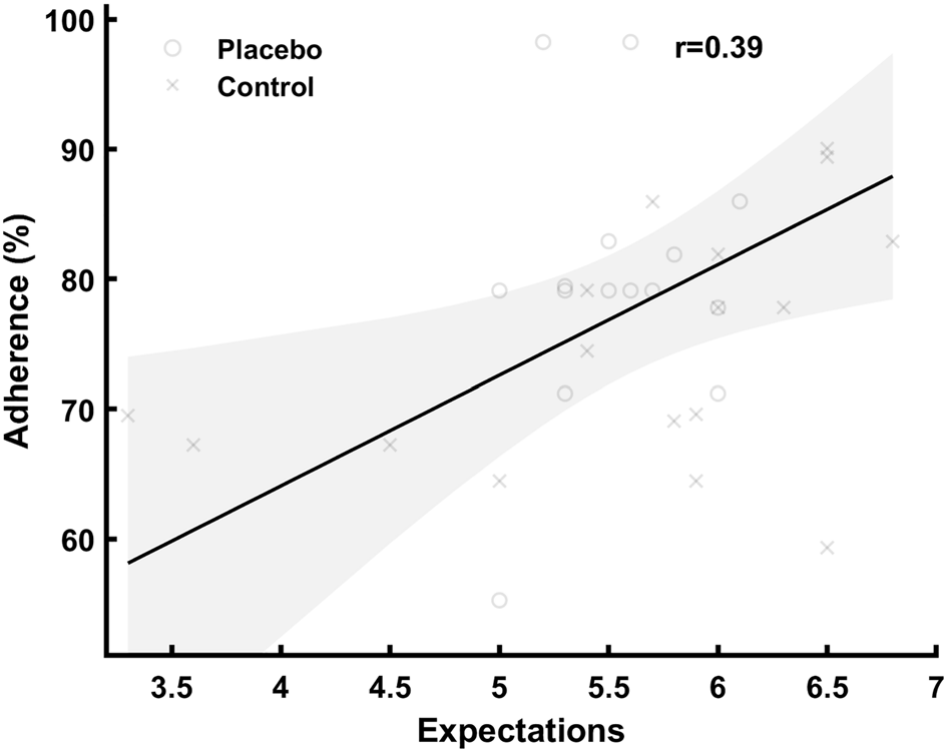
Correlation between the adherence to the training program and expectations toward the training intervention. Adherence is measured as percentage of completed scheduled training sessions and expectation using SETS (Stanford Expectations of Treatment Scale).

Placebo (and nocebo) effects encompass a broad range of events that aren't confined to a direct response to a placebo (or nocebo) treatment^2^. In both a placebo and treatment condition, all the parameters associated with the delivery/engagement of the intervention are included in the results^6^. Expectations, prior experiences, the participant-researcher relationship, trust, empathy, and the ritual surrounding administration are just a few examples^2,5,6^. Those who have attempted to quantify maximal athletic performances, for example, are aware that not all "max" attempts are truly maximal and representational of capacity. As scientists, we usually say things like "subjects were verbally motivated and encouraged to provide their best effort," but what does that really mean? Are we aware of the subjects' pre-conceived thoughts and expectations? Consequently, it is possible that subjects in the placebo group have higher expectations of themselves (or think the researchers expect more of them), and therefore push their limits just slightly more than the subjects that believe they are in the control group^2,5,6^. Such a notion cohere with the results of the present study, as only the 1RM squat showed a significant group difference, where the weight is increased based on participants' and researchers' judgment. Oppositely, the jumping, sprinting, and power tests are slightly less influenced by subjective judgment. Interestingly, although subjective expectations during testing might influence the results, only the placebo group increased muscle thickness, which was not true for the control group. Indicating that there might be part of the placebo effect independent of the testing context. Notably, one should also keep in mind the small sample size and relatively small increase in muscle thickness compared to the measurement error. Another possible explanation for the differences observed is the dissimilarities in expectations towards the intervention, which again predicted the adherence to the training program (Fig. 4). The measure for expectations were non-normal distributed, where it was only a clear difference in expectation for the lower percentile (Fig. 3). The low number of participants and the less sensitive nature of such a questionnaire measure, could be the reason for not observing any stronger difference between groups.

The adherence to the training program appears at first to be an obvious explanation of the group difference in strength gains; however, such a notion is not evident. First, the adherence was not associated with the strength gains (r = − 0.12) nor a significant moderator in an ANCOVA analysis. Therefore, the larger increase in strength in the placebo group seems to be independent of the adherence. Such observation is not surprising, as more training is not always better, especially considering the athletes in the present study were in their competitive season with frequent practice and matches^43^. Secondly, another plausible explanation for the strength gain is higher "quality" training in the placebo group vs. the control group^44^. It is, for example, well documented that the intention to move weights with maximal intentional effort has an impact on training adaptations^44^. Further, it is shown that varying motivational strategies during resistance training influence the exercise performance (i.e., effort/velocity of the movements)^45^. For example, a higher effort is induced by giving athletes feedback during training^45^, creating inter-subject competitiveness^46^, and giving verbal encouragement^47^ among other strategies^48^. It is possible that the subjects in the placebo group performed the training with higher "quality" (i.e., effort) than the subjects in the placebo group, which further influenced the training results. On a similar note, there is also a possibility that the participants in the placebo group might have modified other habits such as sleep or nutrition, or even performed extra exercises outside their allocated program. Unfortunately, due to practical limitations and covid restrictions, we could not supervise and oversee the training sessions or lifestyle habits which could have confirmed or rejected these speculations.

There were no significant differences in any of the included measures regarding the effectiveness of the "individualized training", independent of the placebo group allocation (Table 3). To the authors' knowledge, eight experimental studies have evaluated the effectiveness of individualized training based on force–velocity profiling^13–20^. Four studies did not find any effects in favor of individualized training based on the FV-profile^17–20^. Furthermore, of the four remaining studies, only two included a control group performing a "non-optimized" training regimen for comparison^15,16^. The creators of the concept performed the first of the two studies, where they found large effect sizes from the intervention (ES: 0.7–1.0), with no change in the control group (ES: 0.14)^15,16^. In the latter study by Simpson et al.^15,16^ slightly lower effects were found with effect sizes of 0.37 vs 0.12 for the intervention vs control group, respectively. Notably, according to the original calculations underlying the entire concept of training according to the FV profile, the results from both studies are ~ 5–7 larger than the theoretical framework can account for (Supplement 1). Meaning, that even if one were to accept the hypothesis of individualizing training according to the FV profile, the previous results cannot be explained by the theory alone. Because researchers' and participants' expectations of the intervention can significantly impact the study's outcome, both studies' results are probably confounded by placebo effects^9–11^.

**Table 3:** Results from the sub-groups, strength and power training (BAL) vs high load strength program (STR).

Variable and group	Pre	Post	Change		Group difference			
	Mean ± SD	Mean ± SD	Δ ± SD	ES	Mean ± 95% CI	ES	BF_10_	p-value
1RM squat (kg/bw)								
Strength and power (BAL)	1.61 ± 0.36	1.64 ± 0.31	0.03 ± 0.11	0.09	*BAL vs STR:*			
High load strength (STR)	1.55 ± 0.41	1.64 ± 0.46	0.09 ± 0.10**	0.25	− 0.06 ± 0.08	− 0.16	0.70	0.15
10m sprint (s)								
Strength and power	1.61 ± 0.11	1.60 ± 0.11	− 0.01 ± 0.03	− 0.05	*BAL vs STR:*			
High load strength	1.63 ± 0.11	1.62 ± 0.11	− 0.01 ± 0.03	− 0.12	0.01 ± 0.02	0.06	0.40	0.55
20m sprint (s)								
Strength and power	2.91 ± 0.20	2.89 ± 0.20	− 0.02 ± 0.05	− 0.09	*BAL vs STR:*			
High load strength	2.96 ± 0.20	2.94 ± 0.19	− 0.02 ± 0.07	− 0.11	0 ± 0.04	0.02	0.34	0.88
CMJ Jump height (cm)								
Strength and power	36.6 ± 5.51	37.4 ± 5.8	0.9 ± 1.8#	0.14	*BAL vs STR:*			
High load strength	35.4 ± 7.31	35.7 ± 7.3	0.3 ± 2.1	0.04	0.6 ± 1.54	0.10	0.45	0.39
Leg press power (w/bw)								
Strength and power	20.5 ± 2.7	20.2 ± 2.7	− 0.3 ± 1.3	− 0.12	*BAL vs STR:*			
High load strength	18.7 ± 3.2	18.6 ± 3.3	− 0.1 ± 1.1	− 0.04	− 0.25 ± 0.83	− 0.09	0.37	0.55
Muscle thickness (mm)								
Strength and power	23.5 ± 3.8	23.8 ± 4.2	0.3 ± 2.1	0.08	*BAL vs STR:*			
High load strength	23.6 ± 4.6	24.3 ± 5.1	0.7 ± 1.5	0.18	− 0.4 ± 2.12	− 0.10	0.44	0.63

*1RM* 1-repetition maximum, *CMJ* countermovement jump, *kg* kilogram, *s* seconds, *cm* centimeters, *W* Watts, *mm* millimeters. ***p < 0.001, **p < 0.01, *p < 0.05.

The present study included a large group of trained athletes from handball and soccer, both male and females. Although the experiment was performed as a multicenter study, the same test leaders and equipment were used at all the locations at both testing time points. All the participants were classified as velocity-dominated or well-balanced, resulting in an uneven distribution of participants across the range of FV profiles. As a result, it is impossible to compare smaller subgroups, such as different training regimens for different deficiencies. Importantly, as such uneven allocation is reported in earlier research, the current study was designed with that in mind, where the main analysis is independent of the sub-groups (i.e., both Placebo and Control are on average doing the same type of training). Additionally, the various programs in the subgroups have different training modalities and total volumes calculated as sets × reps, which may influence the sub-group outcomes. The effects we found in the present study were small and would most likely be more prominent if a greater emphasis were placed on inducing a "nocebo" effect in the control group. Nevertheless, as the athletes were in their competitive season, such focus was not regarded as ethical, and it's probably hard to include high-level athletes in such an experiment. Due to the relatively small sample size, the present study should be considered a pilot study, where future full powered trials should be conducted. When interpreting the results from the ultrasound measurements, it is important to consider both the sample size, as well as the observed increases in relation to the measurement error. The small sample size, in combination with a modest increase in muscle thickness, increase the likelihood of random error, making it more difficult to draw definitive conclusions from the data. Similarly, it is worth noting that there was no significant differences between groups in the CMJ, Sprint and leg press measurements. Consequently, there is always a possibility that positive findings in the present study are coincidental and should rather be interpreted together with the broader literature, and not in isolation.

Another limitation in the present study is the lack of tight control of the training sessions performed by the participants. On the other hand, this can also be considered to increase the ecological validity and applicability, as a large portion of training studies are indeed performed under less controlled situations. Finally, we postulated probable mediators of the placebo effect, which should be investigated further in future research to understand the topic better.

## Conclusion

To the author's knowledge, this is the first study to investigate the placebo effect of believing to receive optimal training vs. a generic training program. The results suggest that the placebo effect may explain meaningful outcome variances in sports and exercise training interventions. Future research is needed to better understand the mediators and moderators of the placebo impact on adaptations to training and improvements in sport performance.

## Supplementary Information

Supplementary Information.

## Data Availability

The data that support the findings of this study are available from the corresponding author upon reasonable request.
