# Supplementary material for: The effects of being told you are in the intervention group on training results: a pilot study
Source: Sci Rep. 2023 Feb 3;13:1972. doi: 10.1038/s41598-023-29141-7 (PMC9895971; doi:10.1038/s41598-023-29141-7)
Supplement: Supplementary file 1 — Supplementary Information. [file 41598_2023_29141_MOESM1_ESM.docx]

*Supplementary table 1: Heavy strength training program*

| ***Day 1*** | ***Reps x Set*** | | | | | |  |  |
| --- | --- | --- | --- | --- | --- | --- | --- | --- |
| ***Exercise*** | ***Session 1-3*** | | ***Session 4-6*** | | ***Session 7-9*** | | ***Load/intensity*** | ***Break*** |
| Deadlift | 8 x | 3 | 6 x | 3 | 3 x | 3 | 1-2 RIR | 2-3 min |
| Hip-thrust | 8 x | 3 | 6 x | 3 | 3 x | 3 | 1-2 RIR | 2-3 min |
| Bulgarian split squat | 10 x | 2 | 6 x | 2 | 5 x | 2 | 5-6 RIR | 2-3 min |
| Front squat | 10 x | 2 | 6 x | 2 | 5 x | 2 | 1-2 RIR | 2-3 min |
| Trap bar Deadlift | 5 x | 2 | 5 x | 2 | 5 x | 2 | 70 % 1RM | 3-4 min |
|  |  |  |  |  |  |  |  |  |
|  |  |  |  |  |  |  |  |  |
| ***Day 2*** | ***Reps x Set*** | | | | | |  |  |
| ***Exercise*** | ***Session 1-3*** | | ***Session 4-6*** | | ***Session 7-9*** | | ***Load/intensity*** | ***Break*** |
| Squat | 8 x | 2 | 6 x | 2 | 3 x | 3 | 1-2 RIR | 2-3 min |
| Stiff-leg dead lift | 8 x | 2 | 6 x | 2 | 3 x | 3 | 1-2 RIR | 2-3 min |
| Bulgarian split squat | 8 x | 2 | 6 x | 2 | 5 x | 2 | 5-6 RIR | 2-3 min |
| Trap bar jumps | 5 x | 2 | 5 x | 2 | 5 x | 2 | 50 % 1RM | 3-4 min |
| Calf-raises | 10 x | 2 | 10 x | 2 | 10 x | 2 | 5-6 RIR | 1-2 min |

*RIR= Reps in reserve, 1RM=One repetition maximum, min=minutes, reps=repetitions, Set=training sets.*

*Supplementary table 2: Balanced strength training program*

| ***Day 1*** | ***Reps x Set*** | | | | | |  |  |
| --- | --- | --- | --- | --- | --- | --- | --- | --- |
| ***Exercise*** | ***Session 1-3*** | | ***Session 4-6*** | | ***Session 7-9*** | | ***Load/intensity*** | ***Break*** |
| Deadlift | 8 x | 3 | 5 x | 3 | 3 x | 3 | 1-2 RIR | 2-3 min |
| Front squat | 8 x | 2 | 5 x | 2 | 3 x | 2 | 1-2 RIR | 2-3 min |
| Bulgarian split squat | 8 x | 2 | 7 x | 2 | 3 x | 2 | 5-6 RIR | 2-3 min |
| Hip-thrust | 8 x | 3 | 7 x | 3 | 3 x | 3 | 1-2 RIR | 2-3 min |
| Trap bar jumps | 5 x | 2 | 5 x | 2 | 5 x | 2 | 50 % 1RM | 2-3 min |
| Stair jumps | 5 x | 2 | 5 x | 2 | 5 x | 2 | Bodyweight | 2-3 min |
|  |  |  |  |  |  |  |  |  |
|  |  |  |  |  |  |  |  |  |
| ***Day 2*** | ***Reps x Set*** | | | | | |  |  |
| ***Exercise*** | ***Session 1-3*** | | ***Session 4-6*** | | ***Session 7-9*** | | ***Load/intensity*** | ***Break*** |
| Squat jump w/rubber band | 5 x | 3 | 5 x | 3 | 5 x | 3 | Unloading | 3-4 min |
| Trap bar jumps | 5 x | 2 | 5 x | 2 | 5 x | 2 | 50 % 1RM | 3-4 min |
| Box jumps | 5 x | 2 | 5 x | 2 | 5 x | 2 | Bodyweight | 3-4 min |
| Stair jumps | 5 x | 2 | 5 x | 2 | 5 x | 2 | Bodyweight | 2-3 min |
| Single leg stair jumps | 10 x | 2 | 10 x | 2 | 10 x | 2 | Bodyweight | 1-2 min |
| Deadlift | 8 x | 3 | 6 x | 3 | 4 x | 3 | 1-2 RIR | 2-3 min |

*RIR= Reps in reserve, 1RM=One repetition maximum, min=minutes, reps=repetitions, Set=training sets.*

*Supplementary table 3: “Velocity” training program*

| ***Day 1*** | ***Reps x Set*** | | | | | |  |  |
| --- | --- | --- | --- | --- | --- | --- | --- | --- |
| ***Exercise*** | ***Session 1-3*** | | ***Session 4-6*** | | ***Session 7-9*** | | ***Load/intensity*** | ***Break*** |
| Half Squat | 8 x | 3 | 5 x | 3 | 3 x | 3 | 1-2 RIR | 2-3 min |
| Squat-jumps | 5 x | 3 | 5 x | 3 | 5 x | 3 | Unloading | 3-4 min |
| Trap bar jumps | 5 x | 2 | 5 x | 2 | 5 x | 2 | 50 % 1RM | 3-4 min |
| Explosive Step ups | 5 x | 2 | 5 x | 2 | 5 x | 2 | 10-20kg | 3-4 min |
| Hip-thrust | 8 x | 3 | 5 x | 3 | 3 x | 3 | 1-2 RIR | 2-3 min |
| Countermovement jumps | 5 x | 2 | 5 x | 2 | 5 x | 2 | Bodyweight | 2-3 min |
|  |  |  |  |  |  |  |  |  |
|  |  |  |  |  |  |  |  |  |
| ***Day 2*** | ***Reps x Set*** | | | | | |  |  |
| ***Exercise*** | ***Session 1-3*** | | ***Session 4-6*** | | ***Session 7-9*** | | ***Load/intensity*** | ***Break*** |
| Squat jump w/rubber band | 5 x | 3 | 5 x | 3 | 5 x | 3 | Unloading | 3-4 min |
| Trap bar jumps | 5 x | 2 | 5 x | 2 | 5 x | 2 | 50 % 1RM | 3-4 min |
| Box jumps | 5 x | 2 | 5 x | 2 | 5 x | 2 | Bodyweight | 3-4 min |
| Clean Pull | 5 x | 2 | 5 x | 2 | 5 x | 2 | 50 % 1RM | 3-4 min |
| Stair jumps | 5 x | 2 | 5 x | 2 | 5 x | 2 | Bodyweight | 2-3 min |
| Single leg stair jumps | 10 x | 2 | 10 x | 2 | 10 x | 2 | Bodyweight | 1-2 min |

*RIR= Reps in reserve, 1RM=One repetition maximum, min=minutes, reps=repetitions, Set=training sets.*

Supplementary information:

Each program included written information regarding which group each athlete was allocated to., i.e., Individualized, or control training together with the following information:

*«The program is based on research that shows that it is beneficial to train on the characteristic you are "bad" at. For example, if you performed worst on heavy weights, the program will include more heavy training. Conversely, if you were worst at light weights, the program will contain the most light weights.*

*As this training is part of research, half of the participants are randomly divided into a control group, who receive training regardless of what they are good or bad at. All the training programs will have a beneficial effect, but there is still uncertainty about which is best.»*
